# Impact of smartphone usage on university students' scientific literacy: a mixed-methods approach with importance-performance analysis

**DOI:** 10.3389/fpsyg.2026.1888350

**Published:** 2026-10-19

**Authors:** Jing Luan, Sufei Xin

**Affiliations:** 1 College of Education, Ludong University, Yantai, Shandong, China; 2 Yantai Preschool Education College, Yantai, Shandong, China; 3 Collaborative Innovation Center for the Mental Health of Youth from the Era of Conversion of New and Old Kinetic Energy along the Yellow River Basin, Ludong University, Yantai, Shandong, China; 4 Provincial Experimental Teaching Demonstration Center of Psychology, Ludong University, Yantai, Shandong, China

**Keywords:** attitude, mixed-methods research, scientific literacy, smartphone usage, subjective norms

## Abstract

**Background:**

Previous studies have explored scientific literacy in various educational contexts; however, limited research has specifically examined how smartphone usage is associated with university students' scientific literacy. Given the increasing integration of smartphones into higher education and science learning, it is important to understand the psychological and behavioral mechanisms underlying smartphone use and scientific literacy development. This study aimed to investigate the direct and indirect relationships of subjective norms, perceived behavioral control, attitudes, usage intention, and actual smartphone use with university students' scientific literacy.

**Methods:**

A mixed-methods approach was employed by combining qualitative interviews with quantitative structural equation modeling (SEM) analysis. The study examined the relationships among subjective norms, perceived behavioral control, attitudes, usage intention, actual smartphone usage, and scientific literacy. In addition, an Importance-Performance Matrix Analysis (IPMA) was conducted to identify the most influential predictors of scientific literacy.

**Results:**

The findings revealed that subjective norms and attitudes significantly and positively associated with university students' scientific literacy both directly and indirectly through usage intention and actual smartphone use. Actual smartphone use was also directly and positively associated with scientific literacy, while perceived behavioral control was positively associated with usage intention but showed no significant indirect association with scientific literacy. Furthermore, the IPMA results showed that the importance ranking for predicting scientific literacy was subjective norms, attitudes, actual smartphone use, usage intention, and perceived behavioral control.

**Conclusions:**

Smartphone usage plays a significant role in enhancing university students' scientific literacy when supported by positive attitudes and social influences. The findings provide theoretical support for integrating smartphones into science education and offer practical implications for developing purposeful smartphone-based learning strategies to support scientific literacy among university students.

## 1 Introduction

Nowadays, smartphones have become an indispensable part of daily life for the majority of Chinese university students (99.8% of netizens, of which university students make up 14.2%), influencing their social interactions, entertainment, work, and study habits (Luque-Reca et al., 2024). In this context, the application of smartphones by college students has widely penetrated into many fields such as information acquisition, learning and communication, etc. (Daleiden et al., 2025). Smartphones not only provide a new learning platform for college students by virtue of their powerful information access and rich learning application resources, enabling them to easily access all kinds of academic materials and online courses, but also promote knowledge sharing and cooperation among students through convenient academic communication pathways (Tang et al., 2025; Zhang et al., 2024). It is particularly noteworthy that smartphones have become the main tool of learning for university students, who use them to participate in virtual lectures and online courses, achieving immediacy and flexibility in education (Garzón et al., 2025). This kind of mobile learning not only changes the traditional teaching and learning mode, enabling university students to carry out learning activities anytime and anywhere (Shalini Roy and Gandhimathi, 2025), but also significantly improves the efficiency of learning. Compared to traditional learning materials such as textbooks, the use of smartphones has demonstrated higher learning effectiveness in science education and promoted the development of students' scientific literacy (SL) (Cong and Ironsi, 2025; Garzón et al., 2025). Therefore, smartphones play a crucial role in shaping college students' learning habits and promoting scientific literacy (Durnali, 2025).

The promotion of sustainable development and SL has become a central goal in education globally, highlighting the deep expectations of the world for the scientific competence of the younger generation (Rasa et al., 2023). SL encompasses not only the ability of students to understand and apply scientific concepts to solve problems, but also a deep insight into the nature of science (Almeida et al., 2023), which is crucial for the assessment and application of science and technology (Khishfe, 2023). Against this backdrop, SL has been increasingly discussed by the government and the higher education sector, particularly in the context of science, technology and innovation policies, STEM (science, technology, engineering and maths) education and curriculum reforms. The government has regarded the enhancement of SL for all as a strategic cornerstone of national development, and has strongly promoted the deepening of STEM education through a series of science, technology and innovation policies. Curriculum reform has increasingly emphasized scientific literacy and students' ability to understand and apply scientific knowledge in broader social and educational contexts (Norambuena-Meléndez et al., 2023). As the core carrier of mobile learning, smartphones play an increasingly significant role in promoting SL. It not only provides students with massive learning resources, but also promotes interactive scientific inquiry and deepens students' understanding and application of scientific concepts. The rise of mobile learning mode not only breaks the time and space limitations of learning, but also promotes the development of personalized learning paths, which creates unprecedented opportunities for the comprehensive improvement of SL (Afrina et al., 2023). Therefore, it is particularly important to explore the impact of college students' smartphone use on SL.

Previous literature reveals that SL has been applied in various contexts, including experiential learning (Dopico et al., 2021), multimedia distance learning (Ahied et al., 2020), self-directed learning (Rahmawati et al., 2023), virtual experiments (Liu et al., 2022), and school culture (Prasetyo et al., 2019). In terms of research methods, a variety of techniques have been used in the exploration of SL, including literature analysis (Aristeidou and Herodotou, 2020; Valladares, 2021), interviews (Alneyadi, 2019), meta-analysis (Ho et al., 2019; Nurcahyani et al., 2021), experimental methods (Ahied et al., 2020; Iskandar et al., 2019), and structural equation modeling (SEM) (Guo et al., 2022; Zhu, 2019). For example, Ahied et al. (2020) employed a pre-test and post-test experimental design to investigate the impact of multimedia distance learning on students' SL. Previous studies on SL have not only provided rich information and data revealing the potential and value of SL applications in a variety of contexts such as experiential learning, multimedia distance learning, self-directed learning, virtual experiments and school culture, but also demonstrated a diversity of research methodologies including literature analysis, interviews, meta-analysis, experimental methods and SEM. These research and methodological explorations provide valuable theoretical background and methodological references for this study, and contribute to a more in-depth exploration of how smartphones can be used as a learning tool to promote the development of scientific literacy among university students.

Despite the growing body of research on SL, in-depth studies are scarce on the specific factors affecting the SL of university students in the modern context of smartphone usage. This study addresses this significant gap by extending the research domain of SL to the digital environment, offering a new research avenue for science education. Secondly, to the best of our knowledge, there are no studies to date that have analyzed in depth the importance and performance of the factors influencing scientific literacy among university students. Identifying important factors but relatively low performance can provide detailed practical insights for effective resource allocation management in higher education sectors. Finally, existing studies usually use a single research methodology and rarely use a mixed methodology that combines qualitative and quantitative approaches. This study adopts a sequential mixed-method approach, in which in-depth qualitative analyses are conducted using the interview method, followed by the collection of quantitative data using questionnaires, and the statistical analyses of these data through SEM. This mixed-method approach is able to give full play to the strengths of both qualitative and quantitative research, providing more in-depth and comprehensive explanations and analyses.

The findings from the qualitative phase provided critical insights into the dimensions of smartphone usage and its perceived impact on scientific literacy. These insights informed the formulation of specific research objectives and questions, ensuring they directly addressed the gaps identified in the qualitative analysis. This study aims to explore the mechanisms by which smartphone usage is associated with the SL of university students and to assess the importance and performance of significant predictive factors. It employs a qualitative analysis combined with an SEM to conduct this research. Preliminary qualitative research aims to identify critical factors, guide the survey questionnaire's design for quantitative research, and ensure the accurate capture of core variables. SEM allows for testing the relationships among multiple constructs, which is suitable for revealing the direct and indirect effects between smartphone usage and the development of SL, as well as the importance and performance of significant predictive factors for university students' SL. This mixed-methods approach provides a deeper, integrated perspective on how smartphone usage is associated with SL. Therefore, the research questions are as follows:

(1) What factors influence university students' SL in smartphone usage?
(2) Do these factors affect SL indirectly or indirectly?
(3) What is the order of priority of these factors for improving SL among university students?

The contribution of this research lies in placing the study of SL within the modern context of smartphone usage for the first time and in its pioneering evaluation of the TPB (Theory of Planned Behavior)'s role and applicability in explaining the formation of SL. This not only offers a new perspective on the role of digital tools in education but also adds a new dimension to the theoretical framework of SL. Secondly, the study adopts an innovative mixed methods research design, systematically combining the advantages of qualitative and quantitative methods. More importantly, through this methodological innovation, the study provides a more precise and comprehensive way to identify, analyze, and validate the key factors affecting SL and their interactions. Lastly, this study offers practical and empirical contributions by utilizing the Importance-Performance Matrix Analysis (IPMA) in Partial Least Squares Structural Equation Modeling (PLS-SEM) to evaluate the importance and performance of predictive factors for university students' SL in smartphone usage.

The following is an outline of the structure of this study: The second section provides a review of the literature; the third section outlines the research hypotheses; the fourth section describes the research methodology; the fifth section presents the results derived from data analysis; the sixth section discusses the findings, contributions of the study, limitations, and future directions; and finally, the conclusion of this study is presented.

## 2 Literature review

### 2.1 TPB

The TPB, proposed by Ajzen (1985), aims to predict an individual's specific behavioral intentions (BI) and actual behaviors. TPB consists of five constructs: attitude (ATT), subjective norm (SN), perceived behavioral control (PBC), BI, and actual usage (AU) (Figure 1). According to Ajzen (1991), ATT refers to the degree to which an individual evaluates a behavior as favorable or unfavorable. In this study, ATT refers to the positive or negative evaluation of university students' use of smartphones to learn science; SN pertains to the perceived social pressure to perform or not perform a particular behavior. In this study, SN refers to the social pressure felt by university students when using smartphones to learn science; PBC denotes the degree of subjective control over the behavior itself. In this study, PBC refers to the ease or difficulty of learning science using a smartphone as assessed by university students based on their past experiences and expectations for the future; BI refers to the desire or determination to engage in a specific action. In the present study, behavioral intention is operationalized as usage intention (UI), referring to students' intention to use smartphones for learning scientific knowledge, whereas AU refers to their actual use of smartphones for this purpose.

**Figure 1:**
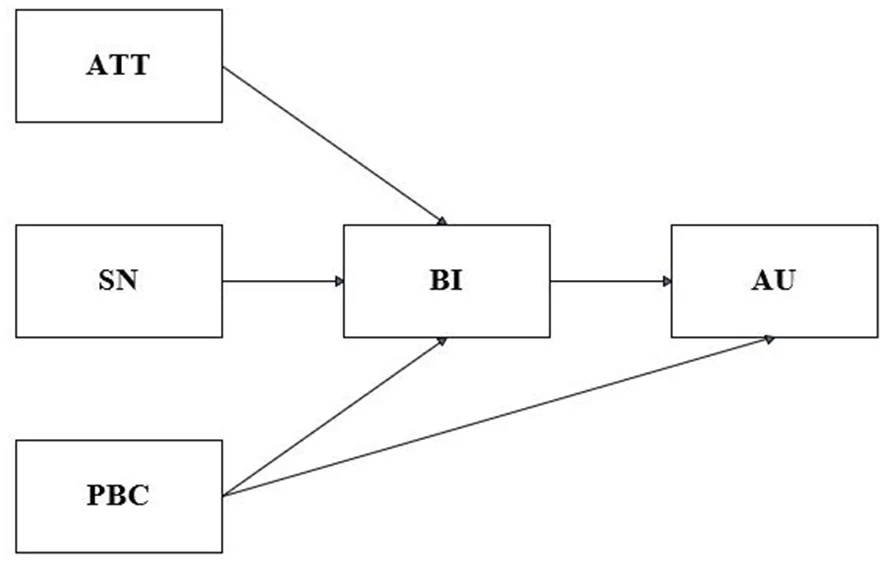
TPB model.

In previous research, the TPB has been widely applied in various contexts to study users' BI, including mobile applications (Ates and Garzon, 2022), massive open online courses (MOOC) (Wang et al., 2020), generative artificial intelligence (Wang et al., 2024), online learning (Li et al., 2022), Facebook use (Raza et al., 2020). These applications are particularly relevant to the present study because TPB has increasingly been used to explain individuals' intentions and behaviors in digital and mobile learning environments. Ates and Garzon (2022), explored the factors influencing Turkish science teachers' intentions to use mobile applications. The results showed that perceived usefulness, perceived entertainment, subjective norms, and perceived behavioral control were significant predictors of usage intention. Similarly, studies of online and mobile learning have shown that attitudes, subjective norms, perceived behavioral control, and behavioral intentions are useful for understanding students' adoption and use of digital learning technologies (Li et al., 2022; Raza et al., 2020). Together, these findings provide empirical support for applying TPB to university students' smartphone-related learning intentions and behaviors.

Accordingly, TPB is used in the present study primarily to explain university students' smartphone-related learning intentions and behaviors rather than scientific literacy itself. Students' evaluations of smartphone-based learning, perceived social expectations, and perceived behavioral control may be associated with their intentions to use smartphones for scientific learning and their subsequent actual usage. These smartphone-related psychological and behavioral processes may, in turn, be associated with students' scientific literacy. TPB therefore provides an appropriate framework for examining the pathways linking smartphone-related learning behavior with self-reported scientific literacy.

### 2.2 SL

SL is the ability of students to engage in discussions on science-related issues and scientific ideas as reflective citizens, and requires them to be able to understand and apply science and technology in a sound manner, which includes understanding phenomena scientifically, evaluating and designing the process of scientific inquiry, and analyzing and interpreting data and evidence scientifically (OECD, 2023). Previous research has identified that factors associated with SL include individual, family, and school factors (Chen et al., 2019; Santhalia and Yuliati, 2021; Tamis-LeMonda et al., 2017; Wen et al., 2020; Xie et al., 2023). Firstly, individual factors affecting SL encompass academic performance (Jufrida et al., 2019), metacognitive reading strategies (Xie et al., 2023) and students' scientific competencies (Shaffer et al., 2019). For example, Shaffer et al. (2019) analyzed data from a SL skills test of 700 university students and found that students' competence and training levels significantly influenced the level of SL. Secondly, family factors influencing SL include parents' education level, occupation (Chen et al., 2019), and the home educational environment (Tamis-LeMonda et al., 2017). Lastly, school factors affecting SL include teaching methods (Wen et al., 2020), learning contexts (Santhalia and Yuliati, 2021), and instructional settings (Alneyadi, 2019). For example, a study by Wen et al. (2020) investigating the impact of interactive simulations as a teaching method on students' SL and school science achievement found that students taught through simulation-guided instructional methods exhibited higher SL compared to those taught through traditional methods.

Scientific literacy has also been assessed using several established instruments, including the Test of Scientific Literacy Skills, Scientific Literacy Assessment, Scientific Inquiry Literacy Test, and Global Scientific Literacy Questionnaire (Mun et al., 2015; Rachmatullah et al., 2016; Shaffer et al., 2019). These instruments reflect different aspects of scientific knowledge, inquiry, reasoning, and individuals' understanding of science.

With the increasing integration of digital technologies into education, research has also begun to examine the relationship between technology use and scientific literacy. For example, Guo et al. (2022) reported an association between ICT use and students' science literacy. More specifically, mobile and smartphone-supported learning has received increasing attention because smartphones provide flexible access to digital resources and allow students to engage in learning activities beyond traditional classroom settings. A recent meta-analysis showed that mobile learning was associated with improved student learning outcomes (Garzón et al., 2025), while mobile-assisted learning has also been examined in relation to self-directed learning (Shalini Roy and Gandhimathi, 2025).

Smartphones are particularly relevant in science education because they can provide immediate access to scientific information, digital learning resources, and interactive learning activities. In a systematic review of smartphone use in science education, documented the increasing integration of smartphones into science-learning activities (Ubben et al., 2023). Similarly, Pramuda et al. (2019) examined smartphone-supported physics learning in relation to students' scientific literacy. These studies suggest that learning-oriented smartphone use may be relevant to students' engagement with and understanding of scientific knowledge.

However, smartphone use is not necessarily beneficial in all learning situations. Research has also drawn attention to smartphone presence and multitasking during studying (Daleiden et al., 2025), as well as problematic smartphone use (Luque-Reca et al., 2024). This distinction highlights the importance of examining learning-oriented smartphone usage rather than treating all forms of smartphone use or screen time as equivalent. Although previous research has demonstrated the educational potential of smartphones, comparatively less is known about how students' attitudes, subjective norms, perceived behavioral control, usage intentions, and actual smartphone use are associated with scientific literacy. The present study addresses this gap by examining these relationships among university students within the TPB framework.

## 3 Research hypotheses

A substantial body of research employing the TPB to explore predictors of AU has shown that UI, ATT, and SN are predictors of AU, while SN, PBC, and ATT are predictors of UI (Usage Intention) (Ates and Garzon, 2022; Bali et al., 2025; Gansser and Reich, 2023; Yang and Xu, 2025; Zhao et al., 2022). (Ates and Garzon 2022) used the TPB to predict factors influencing teachers' use of mobile applications, finding that ATT, SN, and PBC positively correlated with UI, significantly impacting AU. Raza et al. (2020) collected self-reported data from 280 Pakistani university students. They used the PLS-SEM to test the driving factors of university students' use of Facebook, indicating that SN, PBC, and ATT influence the AU of Facebook. Furthermore, the UI of university students using Facebook significantly positively affected AU. This study hypothesizes that the stronger the SN, PBC, and ATT of university students toward smartphones, the more likely they are to influence their UI toward smartphones, thereby increasing AU. Based on the above studies, this research proposes the following hypotheses:

H1: University students' SN toward smartphones significantly influences their UI.
H2: University students' PBC toward smartphones significantly influences their UI.
H3: University students' ATT toward smartphones significantly influences their UI.
H4: University students' UI toward smartphones significantly influences their AU.

Previous literature has indicated that SN and ATT are antecedents of AU (Gong et al., 2025; Saboor et al., 2025; Wang et al., 2025; Wu et al., 2025). Based on TAM and UTAUT2 (Extended Unified Theory of Acceptance and Use of Technology), Wu et al. (2025) found that students' positive attitudes (ATT) toward mobile learning increase perceived usefulness (PU) and Perceived Ease of Use (PEoU), which in turn promote actual use (AU). Drawing on the UTAUT (Unified Theory of Acceptance and Use of Technology) model, Saboor et al. (2025) finds that students' positive attitudes toward online learning technology directly promote actual use and also indirectly do so by strengthening behavioral intention. Meanwhile, Gong et al. (2025) finds that subjective norms significantly and positively influence Chinese fourth-year teacher education students' intention to use LLMs (Large Language Models) for writing, which in turn drives actual use. Based on these findings, this study hypothesizes that the stronger the subjective norms and attitude of university students toward smartphones, the more likely they are to actually use smartphones. Following this rationale, the study proposes the following hypotheses:

H5: University students' SN toward smartphones significantly influences their AU.
H6: University students' ATT toward smartphones significantly influences their AU.

Existing research has found that students' AU of new technologies or systems can enhance their SL (Chen et al., 2025; Liu et al., 2022; Marsuki et al., 2020; Mustikasari et al., 2020; Firdausy and Prasetyo, 2020; Wen et al., 2020). Marsuki et al. (2020) investigated the relationship between secondary students' use of digital learning media and their SL, finding that it contributes to improving students' SL. Firdausy and Prasetyo (2020) reviewed 22 articles published between 2006 and 2018 on the relationship between e-books and SL, concluding that e-books can enhance students' SL. Additionally, preliminary qualitative research indicates a close relationship between individuals' use of smartphones and the improvement of their SL. For instance, Participant 6 mentioned, “Astronomy knowledge apps provide me with a wealth of astronomical data and the latest discoveries, allowing me to access a broader range of astronomical knowledge. Using these apps, I have gained a more comprehensive understanding of the scientific field, which has improved my SL.” Based on these findings, this study hypothesizes that students' AU of smartphones can enhance SL. Therefore, the following hypothesis is proposed:

H7: University students' AU toward smartphones significantly influences their SL.

Multiple studies have confirmed that students' ATT toward the use of new technologies or systems significantly impacts their SL (Ferrer et al., 2022; Jannah et al., 2020; KerŽič et al., 2021; Supriyadi et al., 2023; Suliman et al., 2025; Valverde-Berrocoso et al., 2022). Valverde-Berrocoso et al. (2022) used a literature review to investigate the association between educational technology and students' academic performance, and showed that students who have a positive ATT toward the use of ICTs (Information and Communications Technologies) in the learning process tend to achieve better academic results. That is, when students have a positive perception and use of modern technological tools, their knowledge acquisition, information processing, and problem solving skills are significantly enhanced, thus reflecting a higher level of SL in their academic performance. Ferrer et al. (2022) investigated factors affecting university students' educational engagement, discovering that learning ATT significantly influences learning motivation and engagement, affecting individual's understanding of scientific knowledge and the nature of science. Moreover, in the preliminary qualitative study, many respondents highlighted that an individual's ATT toward smartphones is a catalyst for enhancing SL. For example, one respondent (P1) mentioned, “I have a positive ATT toward using smartphones to learn scientific knowledge. I believe that as long as one has enough self-discipline, they can utilize their phones effectively for learning and enhance their SL.” Based on these findings, this study hypothesizes that university students' positive ATT toward smartphones will enhance SL. Consequently, the research proposes the following hypothesis:

H8: University students' ATT toward smartphones significantly influences their SL.

Subjective norms refer to the perceived social pressure from significant others to engage or not engage in a particular behavior (Ajzen, 1991). Numerous studies have shown that subjective norms positively and significantly influence university students' SL (Miles and Naumann, 2021; Moore and Burrus, 2019). For example, Miles and Naumann (2021) found that the social pressure perceived by university students encourages them to use technological tools such as smartphones more actively, thereby enhancing their SL and self-efficacy. Additionally, research has indicated that SN indirectly improve scientific literacy by increasing students' engagement and scientific identity (Moore and Burrus, 2019). Therefore, this study hypothesizes that the stronger the SN regarding smartphone usage among university students, the higher their SL. Based on the above research, the following hypothesis is proposed:

H9: University students' SN toward smartphones significantly influence their SL.

Based on the above research assumptions, the hypothetical model is proposed in this study (Figure 2).

**Figure 2:**
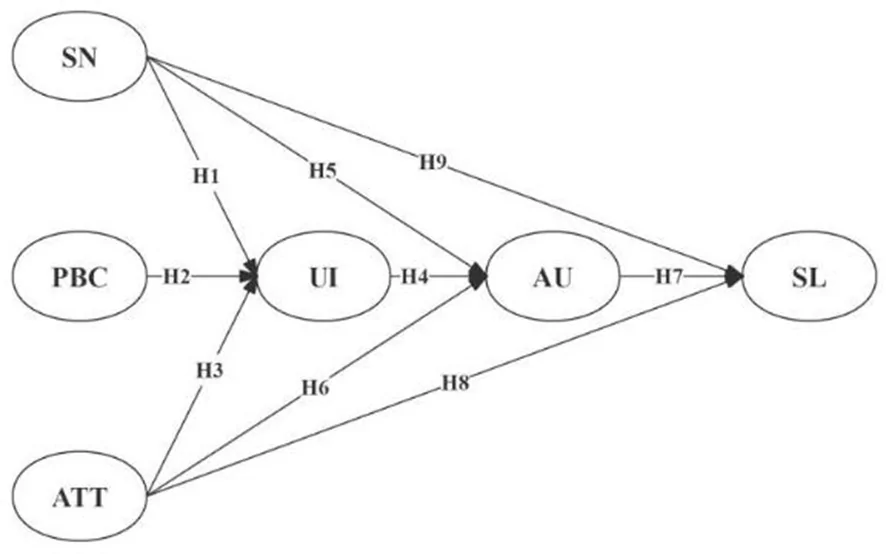
Hypothesis model.

## 4 Methods

### 4.1 Stage 1: preliminary qualitative study

The first phase of this study consisted of a qualitative exploration of university students' experiences of smartphone use in relation to scientific literacy. The qualitative phase was guided by the TPB framework and was designed to examine the contextual relevance of theoretically informed constructs for smartphone-based science learning. The results of the qualitative study were used to screen and contextualize the constructs included in the subsequent quantitative model and to inform the contextual adaptation and organization of the established measures and hypotheses.

#### 4.1.1 Data collection

Semi-structured interviews were conducted with 20 university students (Table 1). The interview protocol was informed by the TPB framework and focused on students' attitudes toward smartphone-based science learning, perceived social influences, perceived behavioral control, intentions to use smartphones for science learning, actual smartphone-use behaviors, and their perceived connections with scientific literacy. Detailed notes were taken during each interview and expanded and organized immediately afterward to ensure accuracy and reduce reliance on memory, thereby reducing potential recall bias (Rubin and Rubin, 2011). The interviews were not audio-recorded. Interview duration was not standardized and varied according to the depth and length of participants' responses. To avoid confounding responses from different interviewees, only one interview was conducted each day and the corresponding interview record was completed and reviewed on that day. This method helped to maintain the integrity of each participant's responses.

**Table 1:** Participants' detail.

Code	Age	Gender	Grade
1	19	Female	Freshman
2	20	Male	Sophomore
3	20	Female	Sophomore
4	19	Female	Freshman
5	21	Male	Junior student
6	22	Female	Senior student
7	21	Female	Junior student
8	19	Female	Freshman
9	20	Male	Sophomore
10	20	Male	Sophomore
11	22	Female	Senior student
12	19	Female	Freshman
13	20	Male	Sophomore
14	20	Female	Sophomore
15	21	Male	Junior student
16	20	Female	Sophomore
17	22	Female	Senior student
18	19	Female	Freshman
19	20	Male	Sophomore
20	21	Female	Junior student

Because the qualitative phase was intended primarily for theory-guided construct screening and contextualization rather than inductive theory generation, no formal thematic-saturation stopping rule was applied. The 20 interviews were used to examine whether the theoretically relevant constructs were consistently represented across participants' accounts.

#### 4.1.2 Data analysis

The qualitative data were analyzed using a theory-informed thematic coding procedure. The TPB provided the initial analytical framework, with ATT, SN, PBC, UI, and AU serving as broad sensitizing categories. Microsoft Word's annotation function was used to code and organize the interview records (Braun and Clarke, 2013). Within these theoretically informed categories, descriptive codes were assigned to specific segments of participants' responses to capture more detailed experiences and perceptions.

As the analysis progressed, the researcher became progressively more selective, focusing on the codes that were most relevant to the research questions. After completing the coding of the first six interviews, a codebook was created to standardize the process and ensure consistency in the application of codes (Braun and Clarke, 2013).

The interview records were then reviewed repeatedly to refine the descriptive codes and examine their fit within the theoretically informed categories. For example, statements about university students' perceptions of smartphone use were coded as “attitudes.” To enhance the credibility and consistency of the coding process, the key points associated with each code were summarized. The researcher manually compared participants' responses within each code to identify similarities, differences, and contextual nuances, including examining the nuances of participants' expressions and the context of their statements. This process also involved addressing any contradictions or conflicts in participants' responses, thereby enriching the thematic analysis by emphasizing the complexity and diversity of respondents' experiences. For example, whilst some participants felt that using a smartphone to learn about science could improve scientific literacy, others expressed how using a smartphone could be a distraction from learning. In addition, a process of constant comparison was employed, whereby each time a new coded segment was flagged, it was compared to the existing codes to optimize the coding and ensure that it accurately reflected the data. Through this iterative process, the researcher merged overlapping codes, refined code definitions and boundaries, and examined the consistency of the theoretically informed categories across participants.

To reduce the influence of the researcher's prior theoretical expectations, divergent and contradictory responses were retained and compared with responses that were consistent with the TPB framework, rather than being excluded from the analysis. The final coding stage examined whether each theoretically relevant construct was meaningfully and consistently represented in participants' accounts and was therefore suitable for inclusion in the subsequent quantitative model.

#### 4.1.3 Results

Finally, the themes were synthesized into a coherent narrative that linked the empirical findings to the research questions and the wider literature. The qualitative analysis showed that the five theoretically informed constructs—ATT, SN, PBC, UI, and AU—were consistently reflected in participants' accounts and were therefore retained for the subsequent quantitative phase. The coding scheme is presented in Supplementary Appendix A.

The qualitative findings were used to establish the contextual relevance of these TPB-related constructs to smartphone-based science learning and to inform the contextual adaptation and organization of the established measures and hypotheses used in Stage 2.

The systematic and comprehensive approach to thematic analysis described above was designed to provide a robust and credible account of participants' experiences and perspectives, contributing valuable insights to the research objectives.

### 4.2 Stage 2: quantitative study

#### 4.2.1 Sample and procedures

All research followed the relevant guidelines and regulations for studies involving human participants, such as the Declaration of Helsinki or similar documents. This study was approved by relevant institutional ethics committee, with approval number LDU-2025-03-0003. Data was collected using the online survey platform Sojump (www.Sojump.com) to test the proposed hypotheses. The data was collected from September 12 to November 21, 2025. Before the survey, participants were fully informed of the study's purpose, assured that their participation was voluntary and that their personal information would be strictly protected. All participants provided informed consent in the form of a written signature.

A non-probability convenience sampling approach with voluntary participation was used. Participants were recruited from four universities located in Shandong, Jiangxi, and Guangxi provinces, including two universities in Shandong, one in Jiangxi, and one in Guangxi. The online survey link was distributed through university and class WeChat groups, and students voluntarily chose whether to participate. Because participants were recruited through accessible university and class groups rather than selected from a complete sampling frame using a probability-based procedure, the final sample should not be interpreted as a random sample.

A total of 406 questionnaires were received. Because the survey link was distributed through university and class WeChat groups and the total number of students who received or viewed the invitation was not recorded, a conventional response rate based on the total number invited could not be calculated.

To ensure sample quality, three criteria were used to screen the questionnaire with reference to Wu et al. (2020). Firstly, the pilot test established that participants generally took 2–5 min to complete the questionnaire. Participants who completed the questionnaire within 90 s were considered to have rushed through the questionnaire in an irresponsible manner and their data were considered invalid. Second, a reverse-coded item was included in the questionnaire, and participants who did not provide a reverse response were considered to have done so, and their data were excluded due to carelessness. Finally, questionnaires with identical responses were also excluded from the analysis. After applying these screening criteria, 36 questionnaires were excluded, leaving 370 valid questionnaires, corresponding to a valid-questionnaire rate of 91.13% among the 406 questionnaires received. The final sample included 101 male participants (27.3%) and 269 female participants (72.7%). The sample was divided by academic year, including 101 first-year students (27.3%), 107 second-year students (28.92%), 107 third-year students (28.92%), and 55 fourth-year students (14.86%). Regarding participants' places of origin, 221 came from rural areas or towns (59.73%), 85 from county-level cities (22.97%), and 64 from prefecture-level cities (17.3%). The educational background of participants' parents was classified as follows: 269 parents (72.7%) had a middle school education or lower, 65 (17.57%) had a vocational college degree, 32 (8.65%) had a bachelor's degree, and 4 (1.08%) had a master's degree or higher. Detailed demographic information is presented in Table 2.

**Table 2:** Demographic information.

Variable	Category	Frequency	Percentage
Gender	Male	101	27.30%
	Female	269	72.70%
Grade	Freshman	101	27.30%
	Sophomore	107	28.92%
	Junior	107	28.92%
	Senior	55	14.86%
Parental education level	Secondary school or below	269	72.70%
	Junior college	65	17.57%
	Bachelor	32	8.65%
	Master or above	4	1.08%

In this study, the maximum number of arrows pointing to endogenous variables was 3. According to Hair et al. (2021), with a significance level of 5% and an *R*^2^ exceeding 10%, the minimum required sample size is 124 participants. This study had 370 participants, thus meeting the minimum statistical requirements.

### 4.3 Measurement

The questionnaire consists of two parts. The first part collects demographic information of the sample. The second part addresses the constructs relevant to this study, See Supplementary Appendix B for the full scale. The scales used in this study were adapted from previous research and modified to fit the context of this study. The ATT scale was developed by Ashrafi et al. (2020) and comprises three items (e.g., “Smartphones are a good tool for learning scientific knowledge”), with a Cronbach's alpha of 0.872. The UI scale was developed by Nathan et al. (2020) and includes three items (e.g., “I will continue to use smartphones to learn scientific knowledge in the future”) with a Cronbach's alpha of 0.883. The SN scale was developed by Sun et al. (2021) and consists of four items (e.g., “Most of my classmates and teachers use smartphones to learn scientific knowledge”), with a Cronbach's alpha of 0.893. The PBC scale was developed by Meng and Choi (2019) and comprises three items (e.g., “I am confident in using smartphones to learn scientific knowledge”), with a Cronbach's alpha of 0.822. The AU scale was developed by Lin (2007) and included three items (e.g., “I frequently use smartphones to learn scientific knowledge”) with a Cronbach's alpha of 0.841. Additionally, to assess the level of SL among university students, a SL instrument adapted from Turgut (2005) was used. In the present study, SL refers to students' self-reported perceptions of their scientific literacy, as the construct was assessed using a Likert-type self-report instrument rather than an objective knowledge or performance test. Therefore, the findings concerning SL should be interpreted as reflecting self-reported scientific literacy rather than objectively demonstrated scientific competence. The original instrument consisted of 38 items covering two theoretical dimensions: Nature of Science (NOS) and Science, Technology, and Society (STS), including 14 NOS items and 24 STS items. The NOS dimension addresses the nature and processes of scientific knowledge (e.g., “Many aspects of our lives cannot be effectively researched using scientific methods”), whereas, the STS dimension concerns the relationships among science, technology, and society (e.g., “Internal social and economic forces within a country have almost no influence on which technologies the country will develop”), with a Cronbach's alpha of 0.922.

During assessment of the PLS-SEM measurement model, the outer loadings of all 38 SL indicators were examined. Using 0.70 as the principal loading criterion during measurement-model refinement, 29 indicators with outer loadings below 0.70 were removed, whereas nine indicators with loadings ranging from 0.702 to 0.790 were retained. Specifically, the retained indicators were SL7 (0.749), SL9 (0.757), SL10 (0.790), SL11 (0.741), SL12 (0.702), SL13 (0.717), SL14 (0.735), SL28 (0.760), and SL37 (0.714). Of these nine retained indicators, seven represented the NOS dimension and two represented the STS dimension. Following the measurement-model refinement, the content validity of the nine retained SL indicators was further evaluated by a panel of 10 experts. All nine indicators met the predefined content-validity criterion, with I-CVI values ranging from 0.80 to 1.00. The overall S-CVI/Ave for the nine-item set was 0.900, with values of 0.914 for the retained NOS indicators and 0.850 for the retained STS indicators. These results provide additional evidence that the retained indicators remained content-relevant and continued to represent both theoretical dimensions of SL.

For transparency, Supplementary Appendix C reports all 38 original SL items, their corresponding theoretical dimensions, outer loadings, and retention or deletion status. Supplementary Appendix D reports the expert content-validity assessment of the nine retained SL indicators. Given the substantial reduction in the number of indicators, particularly within the STS dimension, the retained nine indicators should be interpreted as a narrower empirical representation of SL in the present sample rather than as reproducing the full conceptual breadth of the original 38-item instrument. This study employed a five-point Likert scale for each construct, ranging from (1) strongly disagree to (5) strongly agree.

### 4.4 Data analysis

This study employed a mixed-methods approach, combining qualitative and quantitative methodologies (Venkatesh et al., 2013). Initially, the qualitative method was used to preliminarily identify the factors affecting university students' SL in the context of smartphone usage. This was followed by a survey-based quantitative study to empirically validate the identified relationships. The mixed-methods approach leverages the complementary strengths of qualitative and quantitative methods to provide a more in-depth and detailed explanation of the issue (Venkatesh et al., 2013).

Initially, this study conducted semi-structured interviews with 20 university students to identify the factors influencing their SL in the context of smartphone usage. As a result, five factors were ultimately identified as significant: ATT, SN, PBC, UI, and AU.

Subsequently, a research model was constructed and analyzed using PLS-SEM based on the factors identified in the preliminary qualitative study. PLS-SEM is characterized by the following features (Hair et al., 2019): (1) suitability for small sample studies; (2) capability to estimate complex path models involving multiple latent variables; and (3) focus on the predictive power of the model. Given the preliminary qualitative research outcomes, this study aims to test the predictive ability of a complex hypothesis model comprising six constructs, with the collected data consisting of 370 samples, which is not considered a large dataset. For these reasons, this study chose to use PLS-SEM for data analysis.

## 5 Results

### 5.1 Measurement model

The evaluation of the measurement model primarily involved assessing the reliability and validity of all constructs and their corresponding measurement items. The reliability assessment included the outer loadings, composite reliability, and Cronbach's alpha. As per Byrne (2001), outer loadings above 0.70 indicate high reliability. Twenty-nine indicators of scientific literacy in this study fell below this standard and were therefore excluded, and the nine items retained are detailed in Supplementary Appendix C. Additionally, Cronbach's α ranged from 0.822 to 0.922, meeting the acceptable standard (0.7 is considered the acceptable threshold) (Tavakol and Dennick, 2011). Furthermore, composite reliability between 0.60 and 0.70 is deemed acceptable, while values from 0.70 to 0.90 are generally considered satisfactory (Sarstedt et al., 2021). According to Table 3, all items' composite reliability ranged from 0.828 to 0.924, aligning with the abovementioned standards.

**Table 3:** Reliability and validity.

Constructs	Items	Outer loadings	Cronbach's α	CR	AVE
ATT	ATT1	0.872	0.872	0.874	0.796
	ATT2	0.912			
	ATT3	0.893			
AU	AU1	0.863	0.841	0.842	0.759
	AU2	0.879			
	AU3	0.871			
UI	UI1	0.893	0.883	0.884	0.811
	UI2	0.908			
	UI3	0.901			
PBC	PBC1	0.824	0.822	0.828	0.738
	PBC2	0.895			
	PBC3	0.856			
SL	SL1	0.834	0.922	0.924	0.616
	SL2	0.822			
	SL3	0.794			
	SL4	0.793			
	SL5	0.755			
	SL6	0.769			
	SL7	0.708			
	SL8	0.753			
	SL9	0.83			
SN	SN1	0.864	0.893	0.893	0.757
	SN2	0.887			
	SN3	0.877			
	SN4	0.853			

Convergent validity was assessed using the Average Variance Extracted (AVE). Table 2 indicates that the AVE values ranged between 0.616 and 0.811, surpassing the threshold of 0.5 (Henseler et al., 2016), demonstrating that the convergent validity meets the established criteria.

Discriminant validity refers to the extent to which a construct is distinct from other constructs (Zait and Bertea, 2011). The Fornell–Larcker criterion is used to assess discriminant validity. According to this criterion, a condition for affirming discriminant validity is that the square root of the AVE for each construct should be greater than the correlation coefficients between that construct and all other constructs (Henseler et al., 2016). Based on Table 4, the square root of the AVE for each construct exceeds its highest correlation coefficient with any other construct. Therefore, the results meet the requirements for discriminant validity.

**Table 4:** Discriminant validity (Fornell–Larcker Criterion).

Constructs	ATT	AU	UI	PBC	SL	SN
ATT	**0.892**					
AU	0.742	**0.871**				
UI	0.728	0.771	**0.901**			
PBC	0.73	0.724	0.741	**0.859**		
SL	0.605	0.649	0.616	0.678	**0.785**	
SN	0.699	0.755	0.762	0.766	0.646	**0.87**

The bold value on the diagonal is the square root of AVE.

Additionally, the criteria for assessing cross-loadings indicate good discriminant validity if each item exhibits a higher correlation with its associated latent variable than with other latent variables (Henseler et al., 2016). Table 5 demonstrates that the measurement model possesses satisfactory discriminant validity.

**Table 5:** Discriminant validity (cross-loadings).

Items	ATT	AU	UI	PBC	SL	SN
ATT1	**0.872**	0.640	0.700	0.633	0.550	0.603
ATT2	**0.912**	0.690	0.763	0.653	0.562	0.636
ATT3	**0.893**	0.656	0.751	0.669	0.507	0.632
AU1	0.621	**0.863**	0.669	0.746	0.525	0.628
AU2	0.638	**0.879**	0.687	0.685	0.580	0.646
AU3	0.679	**0.871**	0.659	0.725	0.590	0.696
UI1	0.822	0.686	**0.893**	0.680	0.541	0.641
UI2	0.716	0.702	**0.908**	0.642	0.539	0.675
UI3	0.695	0.694	**0.901**	0.680	0.584	0.743
PBC1	0.594	0.613	0.582	**0.824**	0.583	0.589
PBC2	0.700	0.755	0.682	**0.895**	0.616	0.704
PBC3	0.582	0.747	0.641	**0.856**	0.547	0.676
SL1	0.482	0.518	0.474	0.534	**0.753**	0.508
SL2	0.480	0.515	0.482	0.585	**0.829**	0.515
SL3	0.492	0.526	0.450	0.557	**0.832**	0.534
SL4	0.516	0.570	0.523	0.585	**0.820**	0.580
SL5	0.466	0.499	0.501	0.500	**0.795**	0.483
SL6	0.486	0.543	0.549	0.561	**0.794**	0.517
SL7	0.433	0.512	0.474	0.492	**0.755**	0.518
SL8	0.473	0.472	0.465	0.486	**0.769**	0.473
SL9	0.441	0.415	0.423	0.472	**0.708**	0.418
SN1	0.623	0.654	0.660	0.685	0.557	**0.864**
SN2	0.587	0.660	0.630	0.657	0.546	**0.887**
SN3	0.588	0.625	0.674	0.625	0.562	**0.876**
SN4	0.632	0.686	0.684	0.697	0.581	**0.853**

Bold values indicate the loading of each measurement item on its corresponding intended construct.

### 5.2 Structural model

The structural model analysis in this study encompasses several key aspects: testing for multicollinearity, significance testing, determination coefficient (*R*^2^), mediation effect analysis, and IMPA.

#### 5.2.1 Multicollinearity test

Following the recommendation by Hair et al. (2019), multicollinearity was assessed using the variance inflation factor (VIF) derived from the internal model, with VIF values less than five, indicating that multicollinearity is not a concern. Table 6 indicates that the VIF values for all constructs range from 2.381 to 4.006, all below 5. Therefore, multicollinearity does not pose a problem in this study.

**Table 6:** VIF.

Constructs	ATT	AU	UI	PBC	SL	SN
ATT		3.29	2.381		2.474	
AU					2.94	
UI		4.006				
PBC			2.95			
SL						
SN		2.469	2.695		2.581	

#### 5.2.2 Hypothesis testing

The results of the hypothesis tests are presented in Table 7. Among the direct significant predictors of SL, SN (β = 0.305, *t* = 3.502, and *p* = 0.000) was identified as the most vital determinant, followed by AU (β = 0.286, *t* = 4.069, and *p* = 0.000), and ATT (β = 0.179, *t* = 2.230, and *p* = 0.026). Regarding the direct significant predictors of UI, ATT (β = 0.527, *t* = 9.412, and *p* = 0.000) was the most vital determinant, followed by SN (β = 0.291, *t* = 4.408, and *p* = 0.000), and lastly, PBC (β = 0.134, *t* = 2.186, and *p* = 0.029). Among the direct significant predictors of AU, SN was the strongest determinant (β = 0.358, *t* = 4.740, and *p* = 0.000), followed by UI (β = 0.289, *t* = 3.909, and *p* = 0.000), and lastly, ATT (β = 0.253, *t* = 3.647, and *p* = 0.000).

**Table 7:** Hypotheses testing.

Constructs	β	2.50%	97.50%	*T* statistics	*P*-values	Results
ATT → AU	0.253	0.118	0.392	3.647	0.000	Supported
UI → AU	0.289	0.145	0.434	3.909	0.000	Supported
SN → AU	0.358	0.205	0.504	4.740	0.000	Supported
ATT → SL	0.179	0.021	0.341	2.230	0.026	Supported
AU → SL	0.286	0.147	0.419	4.069	0.000	Supported
SN → SL	0.305	0.143	0.483	3.502	0.000	Supported
PBC → UI	0.134	0.014	0.253	2.186	0.029	Supported
SN → UI	0.291	0.166	0.421	4.408	0.000	Supported

#### 5.2.3 Determination coefficient

The determination coefficient (*R*^2^) is used to measure the extent to which the independent variables explain the variance of the dependent variable. According to Chin (1998), *R*^2^ values can be interpreted as 0.67 (substantial), 0.33 (moderate), and 0.19 (weak). In this study, SL has a moderate determination coefficient (*R*^2^ = 0.491), meaning that all variables explain 49.1% of the total variance in SL (Table 8).

**Table 8:** The predictive power of construct.

Constructs	*R* ^2^
AU	0.681
UI	0.756
SL	0.491

#### 5.2.4 Mediation analysis

Mediation analysis was conducted to the indirect associations of ATT, SN, and PBC with SL through the modeled mediating pathways involving UI and AU. The bootstrap method based on PLS-SEM was used for mediation analysis (Nitzl et al., 2016). Following Sarstedt et al. (2021), the significance of direct and indirect effects evaluate the mediation relationships. Table 9 shows that SN was directly associated with SL (β = 0.305, *p* < 0.001) and also showed a significant indirect association with SL through the modeled pathways involving UI and AU (β = 0.126, *p* < 0.001), indicating partial mediation. Similarly, ATT was directly associated with SL (β = 0.179, *p* = 0.026) and showed a significant indirect association with SL through the modeled pathways involving UI and AU (β = 0.116, *p* = 0.002), also indicating partial mediation. For PBC, the indirect association with SL was not statistically significant (β = 0.011, *t* = 1.473, and *p* = 0.151). Because a direct PBC → SL path was not specified or estimated in the structural model, no conclusion is drawn regarding a direct association between PBC and SL.

**Table 9:** Mediation analysis.

Correlations	Indirect effect	*T*	*P*	Direct effect	*T*	*P*	Type of mediation
SN → SL	0.126	3.782	0.000	0.305	3.502	0.000	PM
PBC → SL	0.011	1.473	0.151	Not estimated	–	–	No significant indirect effect
ATT → SL	0.116	3.118	0.002	0.179	2.230	0.026	PM

FM, full mediation; NM, non-mediation; NA, non-applicable; PM, Partial mediation.

#### 5.2.5 Common method bias

Harman's single-factor test revealed that no single factor accounted for the majority of the variance (Podsakoff et al., 2003). The largest single factor explained only 23.189% of the variance, which is far below the critical threshold of 50% (Podsakoff et al., 2003). Based on the results, this study concludes that there is no significant CMB present.

#### 5.2.6 Importance-performance analysis

To understand the importance and performance of predictors in the model, this study conducted an IPMA (Esmailpour et al., 2020; Varma, 2018). As illustrated in Figure 3, the vertical axis represents performance values, while the horizontal axis indicates importance. The average values for performance and importance were 66.923 and 0.221, respectively. Based on inference, the performance and importance values for the five predictors are as follows: ATT = 68.190 (0.295), AU = 66.249 (0.286), UI = 67.673 (0.083), PBC = 66.838 (0.011), and SN = 65.664 (0.431). The IPMA chart is divided into four quadrants: quadrant 1 in the top right, quadrant 2 in the bottom right, quadrant 3 in the bottom left, and quadrant 4 in the top left (Ooi et al., 2018). Predictors in quadrant 1, such as ATT, have high performance and importance. Predictors in quadrant 2 have low performance but are of high importance and thus require attention, such as AU and SN, followed by those in quadrants 1, 3, and 4 (Varma, 2018). Therefore, AU and SN should be prioritized in this study, followed by UI and PBC.

**Figure 3:**
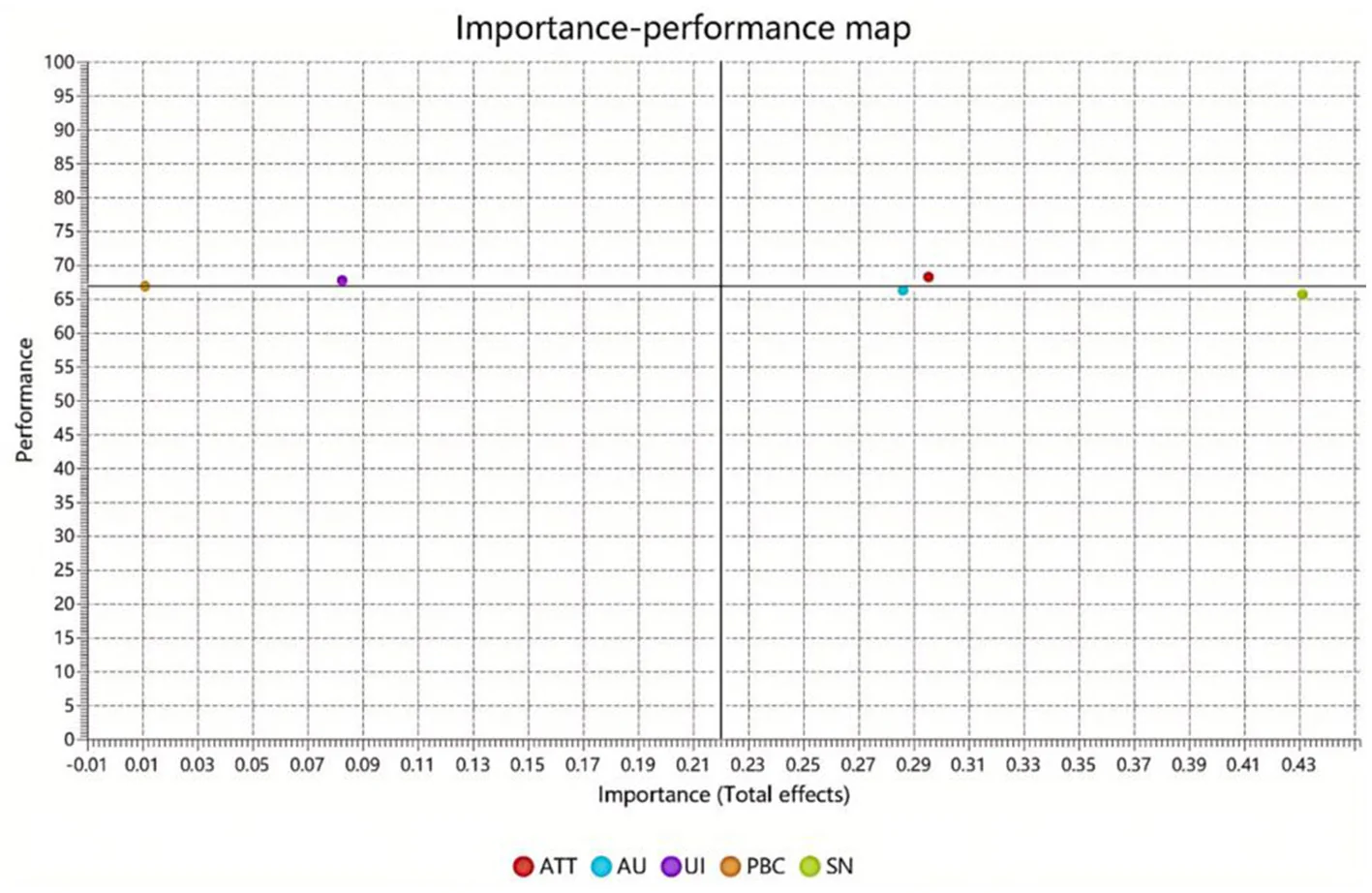
IPMA.

## 6 Discussion

This study aimed to investigate the mechanisms by which smartphone usage is associated with university students' SL. Following a comprehensive review of the existing literature and in the context of smartphone use, this study hypothesized a series of relationships among SN, PBC, ATT, UI, AU, and SL within the TPB framework. The results showed that SN, ATT, and AU were directly and positively associated with SL, while SN and ATT also showed significant indirect associations with SL through the modeled pathways involving UI and AU. PBC was positively associated with UI, whereas its indirect association with SL was not statistically significant. Because a direct relationship between PBC and SL was not specified or estimated in the structural model, no conclusion is drawn regarding a direct association between PBC and SL. The predictive variables collectively explain 49.1% of the total variance in SL. The IPMA further showed that SN had the highest importance for predicting SL, followed by ATT, AU, UI, and PBC. Taken together, these findings indicate that smartphone-related social, attitudinal, intentional and behavioral factors are differently associated with students' self-reported SL.

Among the direct relationships with SL, SN showed the strongest positive association. This finding is broadly consistent with empirical evidence from Turkey, where Ates and Garzon (2022) found that subjective norm was significantly associated with pre-service and in-service science teachers' intentions to use mobile applications for science teaching. Although, their outcome was technology-use intention rather than scientific literacy, their findings highlight the importance of social expectations in educational mobile-technology adoption. The present study extends this evidence by showing that, among Chinese university students, SN was also positively associated with self-reported SL.

A possible explanation can be derived from the normative mechanism of TPB. Students' decisions to use smartphones for scientific learning do not occur in isolation but are embedded in expectations from classmates, teachers, family members, and other significant individuals. When smartphone-based learning is socially recognized as an appropriate and useful academic practice, students may experience greater normative support for using mobile devices to search for scientific information, access learning resources, and participate in science-related learning activities. In this sense, SN may contribute to creating a social environment in which learning-oriented smartphone use becomes more acceptable and more regularly integrated into students' academic routines. This mechanism may help explain why stronger SN was associated with higher self-reported SL in the present sample, supporting H9.

AU was also positively associated with self-reported SL, indicating that students who reported more frequent learning-oriented smartphone use also tended to report higher levels of SL. Relevant empirical evidence has been reported in different geographical contexts. In Indonesia, Pramuda et al. (2019) examined smartphone-supported physics learning and found improvement in specific dimensions of scientific literacy, although the overall difference in global scientific literacy between the experimental and control groups was not significant. In Saudi Arabia, Alsayed et al. (2020) found that undergraduate students extensively used smartphones to access academic information, participate in study groups, and record learning materials. Although the latter study did not directly assess SL, it demonstrates the role that actual smartphone use can play in extending students' access to academic information.

The positive association observed in the present study may therefore be understood in terms of learning opportunities rather than smartphone use itself. Actual learning-oriented use determines whether the technological affordances of smartphones are translated into opportunities to engage with scientific content. Students who regularly use smartphones for science-related learning can access scientific explanations, digital course materials, current scientific information, and other learning resources beyond the temporal and spatial boundaries of the classroom. Such repeated exposure may increase opportunities to interpret and apply scientific information and may therefore be associated with stronger perceptions of scientific literacy. Importantly, because the present study used a cross-sectional design, this finding should not be interpreted as demonstrating that increased smartphone use causes higher SL. Rather, it indicates a positive association between learning-oriented AU and self-reported SL, supporting H7.

ATT was positively associated with self-reported SL, suggesting that students who evaluate smartphone-based scientific learning more favorably also tend to report higher levels of SL. This finding can be compared with empirical research in Palestine, where Salhab and Daher (2023) found that students participating in mobile learning developed more positive attitudes toward learning, with their qualitative findings also highlighting greater motivation, participation, and social interaction. Similarly, research in Turkey found that favorable attitudes were significantly associated with intentions to use mobile applications in science education (Ates and Garzon, 2022). These findings collectively suggest that positive evaluations of mobile technologies are closely connected with students' willingness to incorporate such technologies into educational activities.

From the perspective of TPB, attitude reflects an individual's evaluation of whether a particular behavior is worthwhile or beneficial. In the context of the present study, students who regard smartphones as useful tools for scientific learning may be more willing to devote attention and effort to science-related mobile activities rather than treating smartphones primarily as entertainment or communication devices. Such positive evaluations may encourage more purposeful engagement with scientific resources and make smartphone-based learning a more regular component of students' academic routines. Therefore, the positive association between ATT and self-reported SL may reflect differences in how students perceive and engage with the educational functions of smartphones, supporting H8.

Beyond its direct association with SL, SN also showed a significant indirect association with SL through the modeled pathways involving UI and AU, while the direct relationship remained significant, indicating partial mediation. This pattern provides a more detailed account of how social expectations may be linked to SL. Rather than being associated only with the final learning outcome, SN may first contribute to students' willingness to adopt smartphones for scientific learning and to the extent to which this willingness is translated into actual use. The Turkish evidence reported by Ates and Garzon (2022), in which subjective norm was significantly associated with intention to use mobile applications for science teaching, provides empirical support for the first part of this mechanism. In the present study, SN was significantly associated with both UI and AU, while UI was also positively associated with AU, supporting H1, H4, and H5.

This indirect pathway is theoretically meaningful because social expectations are unlikely to contribute to students' scientific learning merely by existing. Their relevance depends partly on whether students internalize these expectations as behavioral intentions and subsequently engage in learning-oriented smartphone use. For example, encouragement from teachers or peers to use mobile resources for scientific learning may strengthen students' willingness to do so, but any learning-related benefit is more likely to emerge when this willingness is accompanied by actual engagement with scientific content. The partial mediation observed in the present study therefore suggests that the association between SN and self-reported SL involves both a direct social-context component and an indirect behavioral component.

A similar mechanism was observed for ATT. ATT showed a significant indirect association with SL through the modeled pathways involving UI and AU, while its direct relationship with SL remained significant, indicating partial mediation. This suggests that favorable evaluations of smartphone-based learning may be associated with SL not only directly, but also through their relationship with students' intentions and actual smartphone use. Empirical evidence from Turkey similarly shows that positive attitudes are associated with stronger intentions to use mobile applications in science education (Ates and Garzon, 2022), while the Palestinian study by Salhab and Daher (2023) demonstrates the close connection between mobile-learning experiences and favorable learning attitudes. The mechanism underlying this indirect association may lie in the transition from evaluation to intention and then to behavior proposed by TPB. A student may recognize that smartphones provide convenient access to scientific resources, but this positive evaluation becomes educationally relevant only when it is translated into a willingness to use the device for learning and into actual learning-oriented behavior. Among the university students in the present study, a favorable attitude toward smartphone-based scientific learning may therefore increase the likelihood that smartphones are incorporated into routine academic activities, creating more opportunities to encounter and engage with scientific information. This interpretation helps explain why ATT was associated with self-reported SL both directly and indirectly through smartphone-related behavioral processes and supports H3 and H6.

A different pattern emerged for PBC. PBC was positively associated with UI, whereas its indirect association with SL was not statistically significant. This finding is partly consistent with research conducted in Turkey, where Ates and Garzon (2022) found that perceived behavioral control was significantly associated with science teachers' intentions to use mobile applications. Together, these findings suggest that perceiving smartphone-based learning as manageable and within one's control may be particularly relevant to the formation of usage intention, supporting H2.

However, perceived control does not necessarily imply meaningful engagement with scientific content. A student may feel highly capable of operating a smartphone, accessing applications, or locating online information without necessarily using these capabilities for sustained scientific learning. In this sense, PBC may facilitate the formation of an intention to use smartphones, whereas its relevance to SL may depend on whether perceived capability is translated into learning-oriented behaviors of sufficient quality and depth. This distinction provides a possible explanation for why PBC was significantly associated with UI but its modeled indirect association with SL was not significant. Moreover, because a direct relationship between PBC and SL was not specified or estimated in the structural model, the present study does not draw conclusions regarding whether such a direct relationship exists.

The IPMA further indicated that SN had the highest importance for predicting SL, followed by ATT, AU, UI, and PBC. This result reinforces the central role of the social environment observed in the structural model, but it should be interpreted as predictive importance rather than evidence that SN exerts a stronger causal influence than the other constructs. One possible explanation is that learning-oriented smartphone use is socially embedded. University students' perceptions of whether smartphones constitute legitimate learning tools may be shaped by the practices and expectations of classmates, teachers, and other significant individuals. Where such use receives greater social recognition, students may be more willing to integrate smartphones into their learning routines and to engage with scientific content through mobile resources.

The cross-context empirical evidence discussed above also suggests that the relevance of social, attitudinal, and behavioral factors is not confined to a single educational setting. Studies conducted in Turkey, Indonesia, Saudi Arabia, and Palestine have reported associations between mobile technology and students' or educators' intentions, attitudes, learning activities, or science-related outcomes. At the same time, differences in participants, educational systems, and outcome measures mean that these findings should not be treated as directly equivalent to those obtained from the present Chinese university sample. Rather, they provide complementary evidence that helps situate the present findings within a broader international literature. The current study extends this literature by showing how smartphone-related social, attitudinal, intentional, and behavioral factors are associated with self-reported SL among university students in China.

### 6.1 Implications

#### 6.1.1 Theoretical implications

Based on the findings, this study contributes to the existing literature in three theoretical ways. First, by positioning the study of university students' SL within smartphone use, this research provides a new perspective on integrating SL with technology in educational research, pioneering a new domain at the intersection of science education and information technology. This represents the first theoretical contribution of the study.

The second theoretical contribution of this study is adopting a mixed-methods research approach, combining qualitative and quantitative methodologies. Initially, the study utilized qualitative methods to deeply explore influencing factors, establishing a comprehensive research model. Subsequently, quantitative analysis was employed to validate the impact and interrelationships of these factors. Applying this methodological approach enhanced the research's reliability and validity and provided a new path for future studies in similar fields, especially when dealing with complex social science issues.

Through the IPMA, this study highlights the crucial roles of SN, ATT, AU, UI, and PBC in enhancing university students' SL. It identifies SN and AU as the focus areas because both constructs combine relatively high importance with comparatively lower performance in enhancing SL. This suggests that educators and policymakers must provide a healthy, positive, and green social environment for college students' smartphone use and target the efficiency and quality of their smartphone use. This constitutes the third theoretical contribution of this research.

#### 6.1.2 Practical implications

This study delves into the factors associated with university students' SL during smartphone use. The findings hold significant practical implications for enhancing the SL of university students.

This study is the first to introduce university students' smartphone usage into the field of SL research, showing significant positive associations between SN, ATT, AU, and SL. Educators and policymakers should recognize the potential of smartphones as educational tools, especially in supporting university students' SL. This finding suggests that by actively promoting smartphones for scientific learning may be associated with higher levels of students' self-reported SL. Therefore, schools and educational institutions should formulate policies that encourage students to use smartphones responsibly during the learning process and fully leverage technological resources.

This study demonstrates that SN and ATT are directly associated with SL and show indirect associations with it through UI and AU. This implies that when designing interventions to enhance SL, educators should pay particular attention to factors that affect students' intention to use smartphones, such as social pressure and attitudes toward technological tools, to indirectly improve their SL. Additionally, it is crucial to provide students with richer learning resources via smartphones to increase their AU frequency, thus promoting the acquisition and application of scientific knowledge.

The ranking of the predictive factors in this study is as follows: SN, ATT, AU, UI, and PBC. Therefore, in cultivating SL among university students, educators and policymakers should prioritize strategies that focus on adjusting social norms and ATT to guide students' smartphone usage. Specifically, instructional design can promote a positive learning culture by encouraging support for smartphone use from peers, teachers, and parents. This support can influence students' UI and actions, ultimately improving their SL.

### 6.2 Limitations and future work

While this study offers practical and theoretical insights, it also has limitations. Firstly, the quantitative sample consisted of 370 university students from four universities in Shandong, Jiangxi, and Guangxi provinces and was recruited through university and class WeChat groups using convenience sampling. Therefore, self-selection bias cannot be ruled out, and the generalizability of the findings is limited. Future studies should use broader and more representative samples. Secondly, the survey covered 370 college students, which met the sample size requirement needed for the study, however, if a multicohort analysis of grades was done, the fourth year sample did not meet the minimum requirement for statistical sample size and failed to satisfy the requirement for a multicohort professional statistical analysis, which prevented this study from delving deeper into whether or not there were any demographic differences in the impact of college students' mobile phone use on scientific literacy. However, the number of fourth-year students was insufficient for reliable subgroup analysis across academic years. Future research could expand the sample size to fully explore potential differences associated with demographic factors. Furthermore, the original SL instrument contained 38 items, whereas only nine indicators were retained in the final measurement model, with greater representation of the NOS dimension than the STS dimension. Therefore, the final measure may not fully capture the conceptual breadth of the original SL instrument. Future studies should further validate its dimensional structure. In addition, all major constructs were measured using the same self-report questionnaire at a single time point. Although Harman's single-factor test was conducted, common method bias cannot be completely ruled out. Future studies could use multi-source or temporally separated data. Lastly, due to the cross-sectional nature of this study, the observed relationships should not be interpreted as causal effects, and the temporal ordering of the constructs cannot be established. Therefore, future research could adopt longitudinal or experimental designs to examine temporal and potentially causal relationships.

## 7 Conclusion

Based on TPB, this study utilized a mixed-methods approach to investigate how smartphone usage among university students is associated with their SL. Data from 370 university students were collected, and nine hypotheses were tested empirically. The study results indicate that SN, ATT, and AU are directly associated with university students' SL, while PBC is significantly associated with UI. Among these predictors, SN and ATT directly predict SL and show indirect associations with SL through UI and AU. The indirect association between PBC and SL is not statistically significant, while a direct relationship between PBC and SL was not specified or estimated in the structural model. The predictive variables collectively explain 49.1% of the total variance in SL. Regarding importance for predicting SL, the order is as follows: SN, ATT, AU, UI, and PBC. This study provides a new perspective on the role of digital tools in education and adds new dimensions to the theoretical framework of SL. It also offers recommendations for supporting university students' SL from the perspectives of educators and policymakers.

## Data Availability

The raw data supporting the conclusions of this article will be made available by the authors, without undue reservation.
