# Supplementary material for: Impact of smartphone usage on university students' scientific literacy: a mixed-methods approach with importance-performance analysis
Source: Front Psychol. 2026 Oct 19;17:1888350. doi: 10.3389/fpsyg.2026.1888350 (PMC13725464; doi:10.3389/fpsyg.2026.1888350)
Supplement: Supplementary file 1 [file Data_Sheet_1.docx]

**Appendix A. Theory-Informed Coding Scheme and Illustrative Quotations from Interviews**

| **Theoretically informed construct** | **Representative descriptive codes** | **Representative descriptive codes** |
| --- | --- | --- |
| ATT | *“I hold a positive attitude towards using mobile APP to learn scientific knowledge. Although sometimes I will be disturbed by other entertainment applications on mobile phones, I believe that as long as I have enough self-control, I can make good use of mobile apps to learn and improve my scientific literacy”* [P1] | Positive evaluation of smartphone-based learning; perceived usefulness; intuitive presentation of scientific knowledge; diversity of learning resources; distraction concerns |
|  | *“The mobile APP presents scientific knowledge in a way that makes abstract concepts more intuitive and easy to understand. Learning in the form of multimedia makes it easier for me to understand scientific principles and plays a positive role in improving scientific literacy”* [P11] |  |
|  | *“I think there are a variety of learning resources on mobile apps, which can meet my different learning needs. For example, I have a chemistry APP, which has video tutorials of various chemical experiments and the function of simulation experiments. I can choose the corresponding content to learn according to my learning progress and interest, so that I can better understand and master chemical knowledge”* [P9] |  |
| SN | *“My physics teacher recommended a physics learning APP and encouraged us to use it for self-study after class. She said the APP has a lot of practical physics knowledge and exercises to help us better grasp physics knowledge. Thanks to the teacher's recommendation and encouragement, I have a great sense of trust in this APP, and I am more willing to use it to improve my physical literacy”* [P6] | Teacher encouragement; peer influence; parental support; social encouragement to use learning apps |
|  | *“My roommate has been using a biology APP to study. He often shared with me interesting things he had learned in the APP and encouraged me to try it out. Under his influence, I downloaded the APP and started using it to learn about biology. Through communication and sharing with classmates, I became more determined to use mobile APP to learn scientific knowledge”* [P15] |  |
|  | *“My parents always support me to use mobile APP to learn science knowledge. They think it is a very convenient way to learn and can help me improve my learning efficiency. They often encourage me to try different learning apps and discuss the learning content on those apps with me. With their encouragement and support, I became more confident in using mobile APP to learn scientific knowledge”* [P7] |  |
| PBC | *“I have some independent learning ability. When I use the mobile APP to study, I can make my own study plan, arrange the study time, and adjust the study plan in time according to my study situation. For example, when I use a chemistry APP to learn, I will choose appropriate knowledge points for in-depth study according to my learning progress and mastery, and review and consolidate them in time”* [P14] | Self-regulated learning ability; prior scientific knowledge; perceived access to devices and Internet resources |
|  | *“I have some basic knowledge of science. When I use mobile APP to learn scientific knowledge, I can understand and master some basic scientific concepts and principles. For example, when I use a biology APP to learn, I can understand the basic biological knowledge such as the basic structure and function of cells and the basic laws of heredity”* [P8] |  |
|  | *“I have a reliable smartphone and a stable Internet connection, which makes it easy for me to access science education apps anytime, anywhere. This convenience helps me integrate science learning easily into my daily life”* [P20] |  |
| UI | *“Science is an ever-evolving field, with new discoveries and theories emerging all the time. Through the mobile APP, I can get the latest scientific information and research results in time. For example, I will learn about the latest scientific findings through the science news APP, which allows me to keep up with the pace of science and improve my scientific literacy”* [P10] | Willingness to use science-learning apps; perceived value of continued access to scientific information; intention to engage with interactive learning opportunities |
|  | *“Some science apps offer interactive learning features, such as science forums or online LABS. On these platforms, I can communicate and discuss with students and scientists from all over the world and solve problems together. This kind of interactive learning not only expands my scientific vision, but also improves my scientific communication and cooperation ability, which is also an important part of scientific literacy”* [P16] |  |
|  | *“Active use of science education apps can practice my problem-solving skills. By participating in activities such as answering questions and applying knowledge to solve practical problems, I can cultivate deeper scientific thinking and have a positive impact on improving scientific literacy”* [P12] |  |
| AU | *“I often use mobile apps to supplement my classroom learning. For example, when I have a question about a concept in my biology class, I immediately open the biology learning APP to learn more about it. These apps provide intuitive diagrams and detailed explanations to help me understand the points better. This kind of complementary learning anytime and anywhere gives me a deeper understanding of the classroom content and improves my scientific literacy”* [P19] | Actual use for supplementary learning; use of simulation applications; repeated use of science-learning resources |
|  | *“Some interactive functions on mobile apps have made me more interested in science. For example, the Chemistry Experiment simulator APP allows me to perform experiments without real equipment. Through the simulation experiment, I can observe the chemical reaction in person and understand the scientific principles in depth, thus enhancing my interest and curiosity in science”* [P3] |  |
|  | *“Mobile APP provides abundant scientific knowledge resources and helps me expand my knowledge. For example, an astronomy knowledge APP provides me with a wealth of astronomical materials and the latest astronomical discoveries, enabling me to access a wider range of astronomical knowledge. Through the use of these apps, I have a more comprehensive understanding of the field of science, which not only enhances my scientific literacy, but also stimulates my desire to explore unknown areas”* [P6] |  |

Note. ATT, SN, PBC, UI, and AU were used as theoretically informed coding categories based on the TPB framework. Descriptive codes were developed from participants’ accounts within these broader categories. The illustrative quotations show how the constructs were reflected in participants’ experiences and informed their retention in the subsequent quantitative model.

**Appendix B. Scales of all Constructs**

| Constructs | Items | Sources |
| --- | --- | --- |
| SN | 1. Most of my classmates and teachers use smartphones to learn science.  2. Most of my classmates and teachers wish I could use my smartphone to learn science.  3. Most of my classmates and teachers have helped me use my smartphone to learn science.  4. If most of my classmates and teachers use science smartphones to learn science, I will use them too. | Sun et al. (2021) |
| PBC | 1. Whether or not I use my smartphone to learn science is up to me. 2. I am confident in using my smartphone to learn science. 3. I have enough resources, time and opportunities to learn science using my smartphone. | Meng and Choi (2019) |
| ATT | 1. Smartphones are great tools for learning about science. 2. I love using my smartphone to learn about science. 3. I'm eager to use my smartphone to learn science. | Ashrafi et al. (2020) |
| UI | 1. I will continue to use my smartphone to learn science in the future. 2. In the future I will continue to use my smartphone more often to learn about science. 3. I will always use my smartphone to learn science in my daily life. | Nathan et al. (2020) |
| AU | 1. I use my smartphone a lot to learn about science. 2. I prefer to use my smartphone to learn about science. 3. I would recommend the smartphone to a friend to learn about science. | Lin (2007) |
| SL | 1. Scientists may differ in the logical reasoning principles they use to connect evidence and conclusions.  2. Scientists attempt to understand things through new explanations, which sometimes do not fully align with widely accepted scientific theories.  3. A scientific theory should be able to explain evidence or data that were not considered at the time the theory was proposed.  4. Scientific evidence may be distorted during the processes of interpretation, recording, reporting, or data selection.  5. Scientists may emphasize different interpretations of evidence due to their backgrounds, personal beliefs, and values.  6. Scientists attempt to identify potential biases in the work of other scientists.  7. When conducting research, scientists do not assume in advance that they should reach a specific conclusion.  8. In engineering and technology, a design must take all constraints into account (such as physical laws, economic, and political factors). A suitable design achieves the most reasonable trade-off (or balance) among these various constraints.  9. The overall impact of a group’s decision-making—just like the pressure exerted by governments on decisions—can influence the large-scale application of technology. | (Turgut, 2005) |

**Appendix C**

The original Scientific Literacy (SL) scale developed and adapted by Turgut (2005) consists of **38 items** divided into two theoretical dimensions: **Nature of Science (NOS)** and **Science, Technology, and Society (STS)**.

**Note:** The table below lists the complete original 38 items. Rows highlighted in light red are the **9 items retained** in the final measurement model; unhighlighted rows are the 29 items removed during PLS-SEM item purification. (R) = reverse-coded item.

| No. | Dimension | Item Statement (Original English) | Outer loading | status |
| --- | --- | --- | --- | --- |
| 1 | NOS | Scientists examine and evaluate their studies according to certain shared attitudes and beliefs. | 0.687 | Deleted |
| 2 | NOS | There is a belief that the fundamental basis of science is that events in the universe do not occur according to a definite order. (R) | 0.673 | Deleted |
| 3 | NOS | Science assumes that the fundamental rules governing the functioning of nature are the same for the entire universe. | 0.656 | Deleted |
| 4 | NOS | There are many aspects of our lives that cannot be examined through scientific means. | 0.621 | Deleted |
| 5 | NOS | Scientists follow certain procedural steps to reach scientific knowledge without falling into error. | 0.660 | Deleted |
| 6 | NOS | The validity and truthfulness of scientific claims can ultimately be established based on observations. | 0.673 | Deleted |
| ****7**** | ****NOS**** | ****Scientists disagree on the principles of logical reasoning that link evidence and conclusions.**** | 0.834 | Retained |
| 8 | NOS | Putting forward hypotheses and testing hypotheses is one of the most important activities of scientists. | 0.618 | Deleted |
| ****9**** | ****NOS**** | ****Scientists try to understand things by means of new explanations, which are sometimes not fully consistent with the scientific theories that are now widely accepted by everyone.**** | 0.822 | Retained |
| ****10**** | ****NOS**** | ****A scientific theory should be able to account for evidence or data that had not been taken into account at the time the theory was formulated.**** | 0.794 | Retained |
| ****11**** | ****NOS**** | ****Scientific evidence may be distorted in the process of interpreting, recording, reporting or selecting data.**** | 0.793 | Retained |
| ****12**** | ****NOS**** | ****Scientists may emphasise different interpretations of the evidence because of their backgrounds, personal beliefs and values.**** | 0.755 | Retained |
| ****13**** | ****NOS**** | ****Scientists trying to identify possible biases in the work of other scientists.**** | 0.769 | Retained |
| ****14**** | ****NOS**** | ****When conducting research, a scientist does not presuppose that he or she should reach a particular conclusion.**** | 0.708 | Retained |
| 15 | STS | Although science is the pursuit of many different people, it reflects social and cultural values and opinions (e.g., political beliefs, attitudes toward women, etc.). | 0.626 | Deleted |
| 16 | STS | The dissemination of scientific knowledge is not important for the progress of science. (R) | 0.574 | Deleted |
| 17 | STS | Scientific disciplines such as physics, chemistry and biology are separated from each other with definite, clear boundaries. (R) | 0.609 | Deleted |
| 18 | STS | Institutions that provide financial support for research (e.g., various state institutions) guide science (e.g., which research will be conducted). | 0.680 | Deleted |
| 19 | STS | Strong traditions have become established in science; therefore, most scientists behave in accordance with scientific ethical rules in their professional lives. | 0.694 | Deleted |
| 20 | STS | Scientific ethics also concerns the harms that may arise from the application of findings obtained at the end of the scientific research process. | 0.680 | Deleted |
| 21 | STS | Scientific ethics also relates to harms that may arise from scientific experiments. | 0.694 | Deleted |
| 22 | STS | Scientists cannot provide definitive solutions to controversial issues of public concern (e.g., nuclear power or the protection of the environment). | 0.680 | Deleted |
| 23 | STS | New tools and techniques developed together with technology do not contribute much to scientific research. (R) | 0.593 | Deleted |
| 24 | STS | Technology only provides tools and materials for science; technology rarely plays a guiding role in scientific research and theory development. (R) | 0.591 | Deleted |
| 25 | STS | There is no problem that engineers cannot solve. (R) | 0.607 | Deleted |
| 26 | STS | Engineers affect societies and cultures more directly in the short term compared to scientific research. | 0.648 | Deleted |
| 27 | STS | Flawless engineering decisions contain scientific laws. Such decisions also reflect social and personal values at the same time. | 0.686 | Deleted |
| ****28**** | ****STS**** | ****In engineering, a design takes into account all constraints (e.g. laws of physics, economic and political factors). The right design makes the most reasonable compromise (i.e., balance) among the different constraints.**** | 0.753 | Retained |
| 29 | STS | Almost no engineering design is accepted without being tried and tested. | 0.572 | Deleted |
| 30 | STS | Even the smallest technological developments create great effects when they come together. | 0.614 | Deleted |
| 31 | STS | All negative effects that new technological designs may bring about can be predicted in advance. (R) | 0.592 | Deleted |
| 32 | STS | People’s fear of experiences such as flying and driving a car is proportional to the actual dangers they contain. | 0.654 | Deleted |
| 33 | STS | No matter how much money is spent or what measures are taken, there is always a possibility of failure for a technological system. | 0.551 | Deleted |
| 34 | STS | Social and economic forces within a country are influential in determining which technologies will be developed in that country. | 0.417 | Deleted |
| 35 | STS | Technology has very little effect on the nature of societies. (R) | 0.384 | Deleted |
| 36 | STS | In decisions to be made regarding any technology (e.g., building a nuclear power station near a city), only the facts related to that technology are not determinative. | 0.641 | Deleted |
| ****37**** | ****STS**** | ****The overall impact of decisions made by a group of people can affect the large-scale use of science and technology in the same way that government pressure on decision-making can affect the large-scale use of science and technology.**** | 0.830 | Retained |
| 38 | STS | Most decisions on technology-related matters are made without possessing sufficient information. | 0.490 | Deleted |

**Appendix D. Expert Content Validity Assessment of the Retained Scientific Literacy Items**

**D.1 Expert Panel and Rating Procedure**

| Expert ID | Field of Expertise | Academic/Professional Position |
| --- | --- | --- |
| E1 | Science Education | Professor |
| E2 | Science Education | Associate Professor |
| E3 | Curriculum and Instruction | Professor |
| E4 | Educational Measurement | Associate Professor |
| E5 | Educational Psychology | Professor |
| E6 | Educational Technology | Associate Professor |
| E7 | Teacher Education | Professor |
| E8 | Science Education | Associate Professor |
| E9 | Curriculum Studies | Professor |
| E10 | Educational Assessment | Associate Professor |

**Table D1. Expert Panel and Rating Procedure**

| Item | Dimension | E1 | E2 | E3 | E4 | E5 | E6 | E7 | E8 | E9 | E10 | Experts rating 3/4 | I-CVI | Mean rating | SD |
| --- | --- | --- | --- | --- | --- | --- | --- | --- | --- | --- | --- | --- | --- | --- | --- |
| SL7 | NOS | 4 | 3 | 4 | 4 | 3 | 2 | 4 | 3 | 4 | 3 | 9 | 0.9 | 3.4 | 0.7 |
| SL9 | NOS | 4 | 4 | 3 | 4 | 3 | 4 | 4 | 3 | 4 | 4 | 10 | 1 | 3.7 | 0.48 |
| SL10 | NOS | 3 | 4 | 4 | 3 | 4 | 3 | 2 | 4 | 3 | 4 | 9 | 0.9 | 3.4 | 0.7 |
| SL11 | NOS | 3 | 2 | 4 | 3 | 3 | 4 | 2 | 3 | 4 | 3 | 8 | 0.8 | 3.1 | 0.74 |
| SL12 | NOS | 4 | 3 | 4 | 4 | 3 | 4 | 3 | 4 | 4 | 3 | 10 | 1 | 3.6 | 0.52 |
| SL13 | NOS | 3 | 4 | 3 | 2 | 4 | 3 | 4 | 3 | 4 | 3 | 9 | 0.9 | 3.3 | 0.67 |
| SL14 | NOS | 4 | 3 | 4 | 3 | 3 | 4 | 3 | 2 | 4 | 3 | 9 | 0.9 | 3.3 | 0.67 |
| SL28 | STS | 3 | 2 | 4 | 3 | 2 | 4 | 3 | 3 | 4 | 3 | 8 | 0.8 | 3.1 | 0.74 |
| SL37 | STS | 4 | 3 | 3 | 4 | 3 | 2 | 4 | 3 | 4 | 3 | 9 | 0.9 | 3.3 | 0.67 |

Note. I-CVI was calculated as the proportion of the 10 experts assigning a relevance rating of 3 or 4. Thus, an I-CVI of 1.00 represents agreement by all 10 experts, 0.90 represents agreement by nine experts, and 0.80 represents agreement by eight experts.

**Table D2. Summary of Content Validity**

| Dimension | Number of Items | I-CVI Range | Items Meeting Criterion | S-CVI/Ave |
| --- | --- | --- | --- | --- |
| NOS | 7 | 0.80–1.00 | 0.914 | NOS |
| STS | 2 | 0.80–0.90 | 0.850 | STS |
| Overall | 9 | 0.80–1.00 | 0.900 | Overall |

Note. S-CVI/Ave represents the mean I-CVI across the corresponding set of items.

Ashrafi, A., Zareravasan, A., Rabiee Savoji, S., & Amani, M. (2020). Exploring factors influencing students’ continuance intention to use the learning management system (LMS): a multi-perspective framework. *Interactive Learning Environments*, *30*(8), 1475-1497. <https://doi.org/10.1080/10494820.2020.1734028>

Lin, H.-F. (2007). Predicting consumer intentions to shop online: An empirical test of competing theories. *Electronic commerce research applications*, *6*(4), 433-442.

Meng, B., & Choi, K. (2019). Tourists’ intention to use location-based services (LBS). *INTERNATIONAL JOURNAL OF CONTEMPORARY HOSPITALITY MANAGEMENT*, *31*(8), 3097-3115. <https://doi.org/10.1108/ijchm-09-2018-0734>

Nathan, R. J., Victor, V., Tan, M., & Fekete-Farkas, M. (2020). Tourists’ use of Airbnb app for visiting a historical city. *Information Technology & Tourism*, *22*(2), 217-242. <https://doi.org/10.1007/s40558-020-00176-0>

Sun, S., Law, R., Schuckert, M., & Hyun, S. S. (2021). Impacts of mobile payment‐related attributes on consumers' repurchase intention. *International Journal of Tourism Research*, *24*(1), 44-57. <https://doi.org/10.1002/jtr.2481>

Turgut, H. (2005). YAPILANDIRMACITASARIMUYGULAMASININFEN BILGISIOGRETMEN ADAYLARININ BILIMSELOKURYAZARLIK YETERLIKLERINDENSBILIMIN DOGASIVE%BILIM-TEKNOLOJI-TOPLUM ILISKISBOYUTLARININGELISIMINE ETKISi. *YILDIZ TEKNIK UNIVERSITESISOSYAL BILIMLER ENSTITUSU*, 1-260.
